# Application of weighting methods for presenting risk-of-bias assessments in systematic reviews of diagnostic test accuracy studies

**DOI:** 10.1186/s13643-021-01744-z

**Published:** 2021-06-27

**Authors:** Yasaman Vali, Mariska M. G. Leeflang, Patrick M. M. Bossuyt

**Affiliations:** Department of Epidemiology and Data Science, Amsterdam UMC, University of Amsterdam, Meibergdreef 9, 1105AZ, Amsterdam, The Netherlands

**Keywords:** Risk-of-bias assessment, Quality appraisal, Diagnostic accuracy studies, Systematic reviews

## Abstract

**Background:**

An assessment of the validity of individual diagnostic accuracy studies in systematic reviews is necessary to guide the analysis and the interpretation of results. Such an assessment is performed for each included study and typically reported at the study level. As studies may differ in sample size and disease prevalence, with larger studies contributing more to the meta-analysis, such a study-level report does not always reflect the risk of bias in the total body of evidence. We aimed to develop improved methods of presenting the risk of bias in the available evidence on diagnostic accuracy of medical tests in systematic reviews, reflecting the relative contribution of the study to the body of evidence in the review.

**Methods:**

We applied alternative methods to represent evaluations with the Quality Assessment of Diagnostic Accuracy Studies tool (QUADAS-2), weighting studies according to their relative contribution to the total sample size or their relative effective sample size. We used these methods in four existing systematic reviews of diagnostic accuracy studies, including 9, 13, 22, and 32 studies, respectively.

**Results:**

The risk-of-bias summaries for each domain of the QUADAS-2 checklist changed in all four sets of studies after replacing unit weights for the studies with relative sample sizes or with the relative effective sample size. As an example, the risk of bias was high in the patient selection domain in 31% of the studies in one review, unclear in 23% and low in 46% of studies. Weighting studies according to the relative sample size changed the corresponding proportions to 4%, 4%, and 92%, respectively. The difference between the two weighting methods was small and more noticeable when the reviews included a smaller number of studies with wider range of sample size.

**Conclusions:**

We present an alternative way of presenting the results of risk-of-bias assessments in systematic reviews of diagnostic accuracy studies. Weighting studies according to their relative sample size or their relative effective sample size can be used as more informative summaries of the risk of bias in the total body of available evidence.

**Systematic review registrations:**

Not applicable

## Background

Systematic reviews are important tools in evidence synthesis, particularly for combining the results of multiple primary studies which may have conflicting results [1–3]. The credibility of a systematic review depends heavily on the methodological quality of included studies, which impacts the credibility of the findings and the strength of the final conclusions of the review [4]. It is therefore essential that reviewers thoroughly assess the validity of included studies, to appraise the certainty of the evidence in the review and to draw conclusions confidently.

Assessing the risk of bias in primary studies is a fundamental component of systematic reviews. It helps to establish transparency of evidence synthesis results, supports the interpretation of findings and explanations of heterogeneity. Existing guidelines, such as the Cochrane handbook, provide various checklists that can be applied to a diverse array of study designs, for different systematic review types [2, 3, 5–8].

Systematic reviews of diagnostic test accuracy (DTA) studies include evaluations of one or more index tests against a reference standard. Findings from such reviews are used by clinicians when deciding whether a medical test can identify patients with the target condition, or when facing a choice between two alternative tests. However, making a confident clinical decision based on a review of DTA studies can be challenging, since studies included in such reviews may suffer from methodological shortcomings, putting them at risk of bias [8, 9].

The current instrument for evaluating the methodological strength of DTA studies in systematic reviews is known as the Quality Assessment of Diagnostic Accuracy Studies 2 (QUADAS-2) tool. This tool covers four key domains: patient selection, index test, reference standard, and flow of patients through the study and timing of the index test(s) and reference standard [7, 8]. The authors’ final judgments, based on this tool and other instruments, can be presented in reviews as either tables or figures. In Cochrane reviews, these can be created in Review Manager. The two figures that are found most often in systematic DTA reviews as a summary of the risk-of-bias assessment are as follows: a stacked bar chart, showing the proportion of studies with each of the judgments (“Low risk,” “High risk,” “Unclear risk” of bias) and a plot that presents all judgments as a cross-tabulation of studies against domains, usually called a “traffic light” plot [2, 7].

These figures can be presented not only for all studies included in the review but also per meta-analysis specifically. The advantage of presenting traffic light plots alongside forest plots for a specific meta-analysis is that the overall risk of bias for a specific summary estimate can be clear at a glance. Such a summary graph can be regarded as a visual representation of the credibility of the included evidence: the extent to which the included studies are believed to be at low risk of bias. This not only helps the reviewers to consider results of their risk-of-bias assessment when drawing conclusions, it can also help readers, by giving them a quick overview of the validity of the evidence within the review [7, 10]. With a fair and precise presentation of the validity of the studies included in a systematic review, readers will be able to appraise the certainty of the available evidence, a key element for evaluating whether the review findings support a particular clinical recommendation [11]. Cochrane encourages authors to use stratification by overall risk-of-bias judgment as the default strategy in meta-analyses of randomized trials but not for diagnostic test accuracy reviews. An example of a forest plot that displays domain specific risk-of-bias and overall risk-of-bias, with the meta-analysis stratified by overall risk-of-bias, can be seen in a figure presented by Sterne et al. [12].

Studies included in systematic reviews can vary substantially in total sample size and in the relative number of study participants with and without the target condition. These differences will affect summary estimates in meta-analysis, with larger studies typically contributing more to the summary estimates, and studies with more diseased patients having a larger effect on estimates of sensitivity [13–17]. This means that one should be more worried when one of the larger studies in a review is at high risk of bias, compared to a situation in which only a very small study is at high risk of bias. Yet, at present, summaries of risk-of-bias assessments are usually presented at the study level, with all studies contributing in a similar way to such summaries. Although some suggestions were made to use more informative methods of presenting risk-of-bias assessments, which could illustrate the relative contributions of studies with each of risk-of-bias judgment [2, 18], differences in absolute or relative sample size do not seem to be included in the current commonly used method, especially in diagnostic accuracy studies.

We here present alternative methods for summarizing risk-of-bias assessments in systematic reviews of diagnostic accuracy studies. The alternative methods draw more attention to the relative contribution of included studies to the review. By incorporating study sample size or effective sample size in the risk-of-bias summary, rather than just the number of studies, these alternative methods could provide a more informative depiction of the validity of the total body of evidence in the review.

## Methods

### Motivating example

We used existing systematic reviews of diagnostic accuracy studies as examples to illustrate the existing and novel methods of the visual presentation of risk-of-bias. To demonstrate the generalizability of our findings, we selected four reviews that differ in the number of included studies (ranging from 9 to 32), across a variety of clinical domains.

Two systematic reviews targeted non-invasive tests in patients with non-alcoholic fatty liver disease (NAFLD). Studies were eligible if they included adult patients with biopsy-proven or suspected NAFLD for evaluating CK18 [19] or Enhanced Liver Fibrosis (ELF) test [20] as the index test, with liver biopsy as the reference standard. The target conditions were liver fibrosis and non-alcoholic steatohepatitis. One review included 32 reports of studies that had evaluated the diagnostic performance of CK18; the second review summarized 13 studies that had evaluated the ELF test.

The other two selected reviews are Cochrane systematic reviews, published in 2020. One systematic review targeted DTA studies evaluating the performance of measured hippocampal volume with structural magnetic resonance imaging for the early diagnosis of dementia due to Alzheimer’s disease in people with mild cognitive impairment. Twenty-two studies were included in this systematic review [21]. The fourth systematic review aimed to assess the diagnostic accuracy of transcranial Doppler and transcranial color Doppler for detecting stenosis and occlusion of intracranial large arteries in people with acute ischemic stroke. This study included 9 DTA studies [22].

### Reporting risk-of-bias assessment methods

The risk-of-bias assessment results of the four systematic reviews are presented in tables and illustrated in figures, using the current method and two alternative methods to show how the implementation of the new methods can alter the overall risk-of-bias assessment summary.

In all selected systematic reviews, two reviewers had used the QUADAS-2 tool to assess risk-of-bias and concerns about the applicability in the studies. In this report, we do not discuss possible consequences of our method for the concerns regarding applicability. We believe that applying these alternative methods to the risk-of-bias part of the four domains of QUADAS-2 tool could sufficiently illustrate the potential differences between the respective methods.

#### Current method

Using the commonly used risk-of-bias method, we generated bar graphs that display the proportion of studies with each of the risk-of-bias judgments for each of the four domains of the QUADAS-2 tool.

#### Weighted method—sample size

The commonly used risk-of-bias assessment and summary figures rely on the number of studies at the respective levels of risk-of-bias in each domain. This ignores the relative size of the included studies in the total risk-of-bias assessment. A study with a relatively large sample size contributes more to the review but is treated equally, compared to a study with a much smaller sample size.

The Cochrane handbook for systematic reviews of interventions recommends to present the risk-of-bias assessment results by restricting attention to studies in a particular importance to meta-analysis and to represent the proportion of information at different risk-of-bias levels [2]. However, such weighted plots are not producible in Cochrane’s Review Manager.

It is very well possible to assign different weights to the studies when preparing summaries, to display how the included studies contribute to the total body of evidence in the review. One way to do so is using relative total sample size as the weight, which reveals the relative contribution of each study to the total group of patients for which data are included in the systematic review. Assigning differential weights to studies based on their relative sample size would be especially influential when considerable differences in sample size exist between included studies.

Accounting for differences in sample size in risk-of-bias assessment would bring this step of systematic reviews in line with methods for meta-analysis, which do not rely on vote counting on a study-by-study level, but incorporate the relative precision of each study in producing summary estimates. In general, recommended methods include inverse variance-weighted average methods or relying on weighted sums of z-scores [13]. Similar to these weighting methods for interventional studies, weighted average estimators are presented for meta-analysis of diagnostic test accuracy studies [23]. In DTA reviews, hierarchical methods, such as the bivariate logit-normal model, also account for between-study differences in sample size [24, 25].

#### Weighted method—effective sample size

Simple weighting by sample size may not be always sufficient [16, 17]. Study groups that are equal in size can include quite different numbers of participants with (n2) and without the target condition (n1). The proportion of cases with the target condition commonly differs across the various setting accuracy studies are conducted in. Consequently, these differences can affect the precision of an estimate of test accuracy for a given total sample size [16, 23].

An alternative is to rely on the effective sample size as a more appropriate method to display the relative contribution of a study. Deeks and his colleagues presented a simple formula for calculating effective sample size in DTA studies and stated in their report that “sample size related precision when there are unequal group sizes is more appropriately summarized by the effective sample size, where ESS= (4n1n2)/(n1 + n2)” [16].

After presenting the findings of the four systematic reviews based on the current risk-of-bias assessment method and the proportion of studies at low, unclear, and high risk of bias, we then used our new methods and replaced the proportion of studies with total sample size of individual studies and their effective sample size at different risk-of-bias levels [16]. Accordingly, we presented an alternative version of the graphs to present the summary, one that relies on the sample size and effective sample size of the included studies at different levels of risk-of-bias.

## Results

**Fig. 1:**
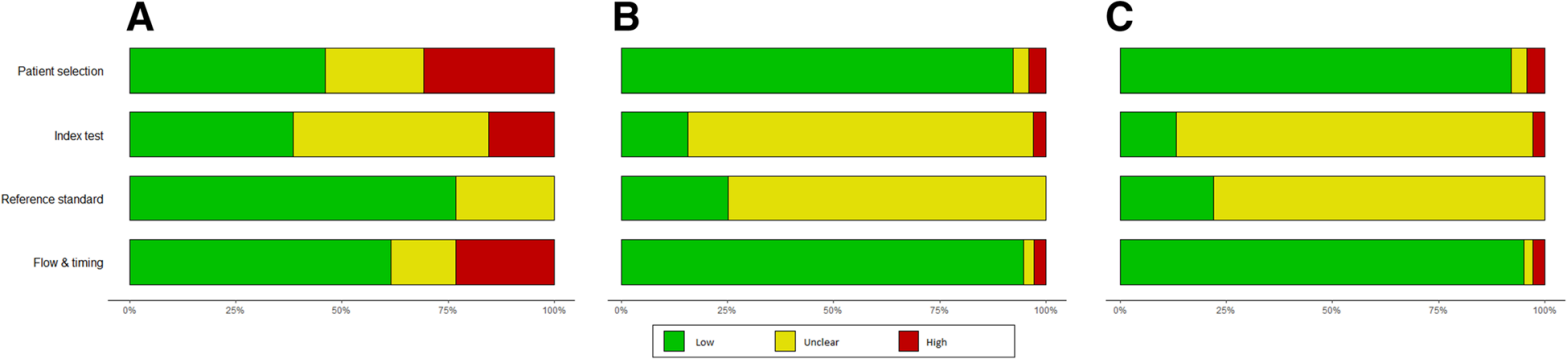
Results of risk-of-bias assessment plots, which illustrate the judgments (“Low risk,” “High risk,” and “Unclear risk” of bias) for four QUADAS-2 tool domains (x-axis) based on (**A**) proportion of included studies, (**B**) proportion of included patients (**C**) effective sample size of 13 included studies in the ELF systematic review (y-axis)

**Fig. 2:**
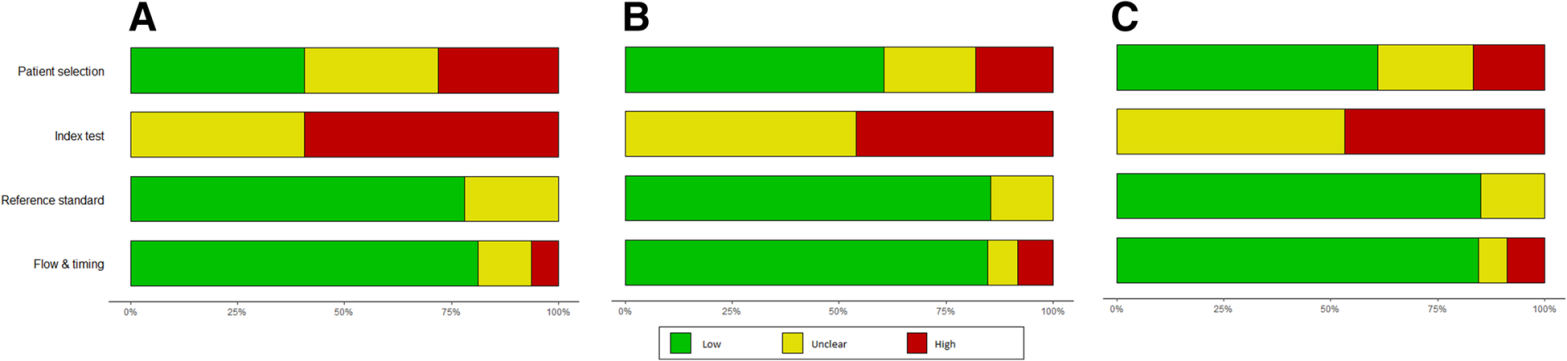
Results of risk-of-bias assessment plots, which illustrate the judgments (“Low risk,” “High risk,” and “Unclear risk” of bias) for four QUADAS-2 tool domains (x-axis) based on (**A**) proportion of included studies, (**B**) proportion of included patients (**C**) effective sample size of 32 included studies in the CK18 systematic review (y-axis)

**Fig. 3:**
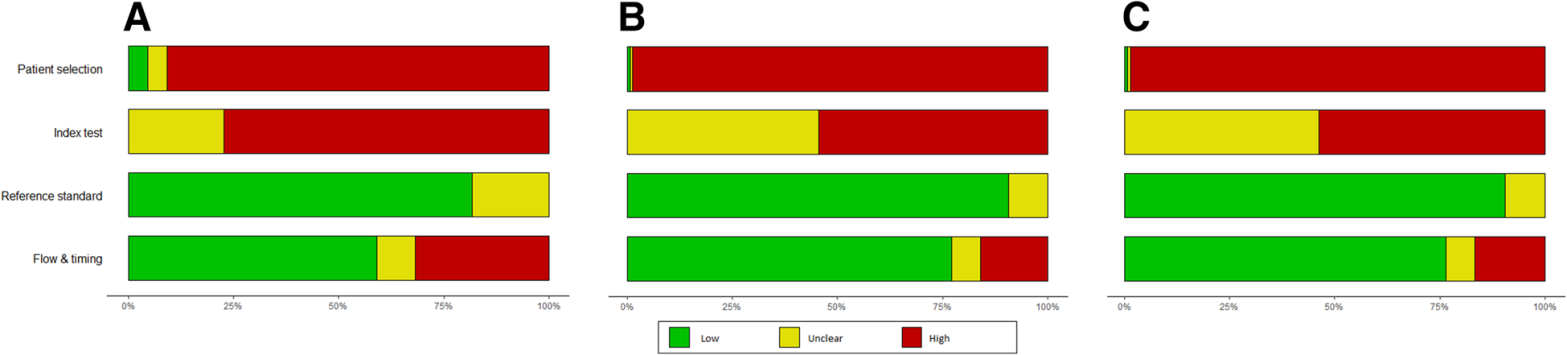
Results of risk-of-bias assessment plots, which illustrate the judgments (“Low risk,” “High risk,” and “Unclear risk” of bias) for four QUADAS-2 tool domains (x-axis) based on (**A**) proportion of included studies, (**B**) proportion of included patients (**C**) effective sample size of 22 included studies in the Lombardi 2020 systematic review (y-axis)

**Fig. 4:**
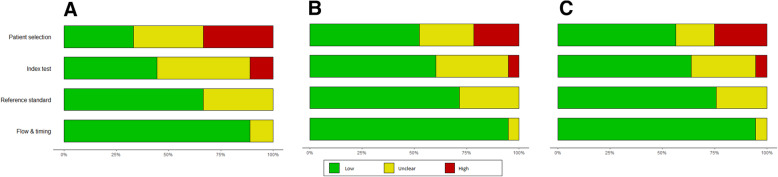
Results of risk-of-bias assessment plots, which illustrate the judgments (“Low risk,” “High risk,” and “Unclear risk” of bias) for four QUADAS-2 tool domains (x-axis) based on (**A**) proportion of included studies, (**B**) proportion of included patients (**C**) effective sample size of 9 included studies in the Mattioni 2020 systematic review (y-axis)

The results of the current risk-of-bias assessment method are presented in Figs. 1, 2, 3, and 4A, which illustrate the proportion of studies at different risk levels. While the findings from the alternative weighting methods are illustrated in Figs. 1, 2, 3, and 4B and C. In the tables, we reported the findings as frequency and percentage of low, unclear, and high risk of bias for each QUADAS-2 tool domain.

Figure 1A shows the summary risk-of-bias plot of studies that evaluated the performance of the ELF test in detecting liver fibrosis or NASH in NAFLD patients. This summary plot is based on the percentages of included studies. In contrast, Fig. 1B and C shows the assessment results when including study sample size or effective sample size, respectively. For the patient selection domain, the risk of bias was high in 31% of studies. However, after replacing the number of studies with the relative sample size and effective sample size of the individual studies, it changed to significantly smaller proportions (4%). The results in unclear and low-risk levels also changed when using alternative weighted methods: 23% vs 4% and 46% vs 92%.

In the other domains, a similar, considerable difference was observed between the results of non-weighted and weighted methods. For instance, in the index test domain, the percentage in the high-risk level changed from 15 to 3%. In the unclear and low-risk levels of this domain, differences were observed not only between the current risk-of-bias and the weighted methods but also between the two-weighted methods. The results changed from 46 to 38% in the first assessment to 81% and 16% using sample size, and from 84 to 13% when relying on effective sample size weighting method.

In the reference standard domain, there were no studies at high risk-of-bias. The 23% of studies for which risk-of-bias level was judged “unclear” changed to 75% of patients, after applying weights based on sample size. While at low risk-of-bias, the number changed from 77% of studies to 25% of population. The effective sample size weighting method resulted in 78% and 22% at unclear and low risk-of-bias, respectively.

The results in the flow and timing domain also changed from 23 to 3% in high-risk level, from 15 to 2% in unclear-risk level and from 62 to 95% in low-risk level after applying weights to the studies. See Table 1 for the details.

**Table 1:** Risk-of-bias (RoB) levels based on proportion of studies, their sample size, and effective sample size in ELF systematic review

QUADAS2 tool domain	RoB based on number of studies			RoB based on sample size			RoB based on effective sample size		
Risk of bias	Low	Unclear	High	Low	Unclear	High	Low	Unclear	High
Patient selection	46%	23%	31%	92%	4%	4%	92%	4%	4%
Index test	38%	46%	15%	16%	81%	3%	13%	84%	3%
Reference standard	77%	23%	0%	25%	75%	0%	22%	78%	0%
Flow and timing	62%	15%	23%	95%	2%	3%	95%	2%	3%

Using different weighting methods also showed noticeable changes in risk-of-bias assessment results for the other selected systematic reviews. See Figs. 2, 3, and 4 for the risk-of-bias summary plots before weighting (A) and after using weighted methods based on sample size (B) and effective sample size (C). Tables 2, 3, and 4 show the detailed changes in percentages of each level of bias in different QUADAS-2 domains. In general, the observed differences between the methods were more noticeable when the reviews included a smaller number of studies with wider range of sample size.

**Table 2:** Risk-of-bias (RoB) levels based on proportion of studies, their sample size, and effective sample size in CK18 systematic review

QUADAS2 tool domain	RoB based on number of studies			RoB based on sample size			RoB based on effective sample size		
Risk of bias	Low	Unclear	High	Low	Unclear	High	Low	Unclear	High
Patient selection	41%	31%	28%	60%	22%	18%	61%	22%	17%
Index test	0%	41%	59%	0%	54%	46%	0%	53%	47%
Reference standard	78%	82%	0%	85%	15%	0%	85%	15%	0%
Flow and timing	81%	13%	6%	85%	7%	8%	85%	7%	9%

**Table 3:** Risk-of-bias (RoB) levels based on proportion of studies, their sample size, and effective sample size in Lombardi 2020

QUADAS2 tool domain	RoB based on number of studies			RoB based on sample size			RoB based on effective sample size		
Risk of bias	Low	Unclear	High	Low	Unclear	High	Low	Unclear	High
Patient selection	5%	5%	91%	1%	1%	99%	1%	1%	99%
Index test	0%	23%	77%	0%	46%	54%	0%	46%	54%
Reference standard	82%	18%	0%	91%	9%	0%	90%	10%	0%
Flow and timing	59%	9%	32%	77%	7%	16%	77%	7%	17%

**Table 4:** Risk-of-bias (RoB) levels based on proportion of studies, their sample size, and effective sample size in Mattioni 2020

QUADAS2 tool domain	RoB based on number of studies			RoB based on sample size			RoB based on effective sample size		
Risk of bias	Low	Unclear	High	Low	Unclear	High	Low	Unclear	High
Patient selection	33%	33%	33%	52%	26%	22%	56%	18%	25%
Index test	44%	44%	11%	60%	35%	5%	64%	31%	5%
Reference standard	67%	33%	0%	72%	28%	0%	76%	24%	0%
Flow and timing	89%	11%	0%	95%	5%	0%	95%	5%	0%

## Discussion

We presented alternative methods to summarize the risk-of-bias assessments in systematic reviews of diagnostic test accuracy studies. By using these methods, including either relative sample size or relative effective sample size of the individual studies, we observed considerable visual changes for the four examples when presenting the risk-of-bias levels for each domain of the QUADAS-2 checklist, compared to the common unweighted method, which relies on the proportion of studies.

Systematic reviews and meta-analyses have become increasingly important in healthcare settings. Policy makers and clinicians rely on high quality systematic reviews for their decision-making. Yet, as a form of observational research, systematic reviews are susceptible to potential bias. When some of the included studies have methodological shortcomings, the meta-analytic results may be jeopardized [26, 27]. As studies included in a systematic review can be heterogeneous, also in terms of methodological rigor, they can, could, or should contribute in a different way to the total body of evidence, depending on their strengths and weaknesses [28].

Scores resulting from the risk-of-bias assessment could be used to weight the data of different studies included in a meta-analysis [29]. Work has been done in DTA systematic reviews on different methods of weighting studies according to their quality assessment result, to produce different risk-of-bias summaries, or to incorporate these in meta-analysis [30]. However, a common criticism of this approach is the lack of an empirical basis for deciding how much weight to assign to different domains of bias [2, 17, 31]. It has also been argued that calculating a summary score could lead to questionable assessments of validity [32] and that such scales may be less likely to present transparent summaries for review readers. For this reason, methodologists recommend avoiding direct weighting of effect estimates by risk-of-bias assessment results [2, 31].

We believe that meta-analysis is not the only phase in a systematic review that requires careful consideration of differences between included studies. Incorporating the methodological strength of the included studies in reports of reviews can and should influence conclusions drawn from the reviews. In a systematic review that included studies of different sizes and with methodological differences, studies that differ in their risk of bias should contribute differently to the total body of evidence. In our study, applying the alternative weighting methods illustrated how one large study at high risk of bias can be more influential in the total risk-of-bias assessment than a tiny study, also at high risk of bias. We believe methods for presenting risk-of-bias judgments that incorporate study weights can provide both authors and readers with more informative results of the risk-of-bias assessment. This will help in building valid conclusions and can facilitate decision-making based on the review findings.

Primary studies in a single systematic review may also have been performed in different settings and populations, with consequences for disease prevalence, even for studies with an identical sample size. Subsequently, differences in the relative balance of diseased and non-diseased study participants can affect precision of the accuracy estimates, for a given total sample size. Although we observed only small differences between total and effective sample size methods in our selected examples of systematic reviews, we believe that relying on effective sample size in summarizing risk-of-bias assessments, rather than on total sample size can be an even more informative weighting method, especially when the number of included primary studies is small and disease prevalence varies substantially [20, 22].

To facilitate the production of risk-of-bias assessment figures, a new Risk-Of-Bias VISualization tool, robvis, has recently been presented as an R package and a web app [18]. In this platform, a measure of the precision of the estimate, such as the weight assigned to that result in a meta-analysis or the study sample size, can be included to create the summary risk-of-bias plot. At present, the package cannot yet produce graphs that show applicability concerns. Modifying bias domains within the tools is only possible for the “ROB1” option, which can handle varying numbers of columns, since authors using this tool frequently add or remove bias domains within this tool. Moreover, it is important to know how much awarding weights to the studies changes the risk-of-bias assessment findings, as in some levels the difference might be small and not recognizable in plots. We believe that the package could be further improved, providing percentages in the risk level at each domain, thereby helping authors in comparing weighted and unweighted methods and in interpreting the findings correctly.

Our examples were based on the QUADAS-2 risk-of-bias assessment tool for test accuracy studies. Future research could explore other risk-of-bias tools, as well as the impact on reviews with different levels of heterogeneity in included studies. It would also be informative to explore systematically to what extent systematic review authors and readers respond to these new weighted methods of risk-of-bias assessment.

## Conclusion

We here have shown that an alternative way of summarizing risk-of-bias assessments with the QUADAS-2 tool can be used, one that does more justice to the relative contribution of each study to the total body of evidence included in the review. This can be achieved by using weights, either based on sample size or on effective sample size. We recommend reviewers select one of these alternative methods of weighting for summarizing the risk-of-bias assessment and to pre-specify the selected approach in the systematic review protocol, to avoid potential bias.

Evaluating and reporting the risk of bias in a review, thereby informing the readers about the limitations in the available body of evidence, will not be sufficient to produce valid conclusions. We call on reviewers to also incorporate the risk-of-bias assessment into their interpretation of the available data, their conclusions, and in the summary of findings. Only then we can trust that the conclusions in the review do justice to the validity of the research findings included in the systematic review.

## Data Availability

The datasets used and/or analyzed during the current study are available from the corresponding author on reasonable request.

## Abbreviations

DTA: Diagnostic Test Accuracy
QUADAS-2: Quality Assessment of Diagnostic Accuracy Studies 2
NAFLD: Non-Alcoholic Fatty Liver Disease
ELF: Enhanced Liver Fibrosis
RoB: Risk of Bias
ESS: Effective Sample Size
NASH: Non-Alcoholic Steatohepatitis
